# Comparative Evaluation of Direct Thrombin and Factor Xa Inhibitors with Antiplatelet Agents under Flow and Static Conditions: An *In Vitro* Flow Chamber Model

**DOI:** 10.1371/journal.pone.0086491

**Published:** 2014-01-31

**Authors:** Kazuya Hosokawa, Tomoko Ohnishi, Hisayo Sameshima, Naoki Miura, Takehiko Koide, Ikuro Maruyama, Kenichi A. Tanaka

**Affiliations:** 1 Research Institute, Fujimori Kogyo Co., Yokohama, Kanagawa, Japan; 2 Department of System Biology in Thromboregulation, Graduate School of Medical and Dental Sciences, Kagoshima University, Kagoshima, Japan; 3 Joint Faculty of Veterinary Medicine, Kagoshima University, Kagoshima, Japan; 4 Department of Anesthesiology, Vascular Medicine Institute, University of Pittsburgh Medical Center, Pittsburgh, Pennsylvania, United States of America; Aligarh Muslim University, India

## Abstract

Dabigatran and rivaroxaban are novel oral anticoagulants that specifically inhibit thrombin and factor Xa, respectively. The aim of this study is to elucidate antithrombotic properties of these anticoagulant agents under arterial and venous shear conditions. Whole blood samples treated with dabigatran or rivaroxaban at 250, 500, and 1000 nM, with/without aspirin and AR-C66096, a P_2_Y_12_ antagonist, were perfused over a microchip coated with collagen and tissue thromboplastin at shear rates of 240 and 600 s^−1^. Fibrin-rich platelet thrombus formation was quantified by monitoring flow pressure changes. Dabigatran at higher concentrations (500 and 1000 nM) potently inhibited thrombus formation at both shear rates, whereas 1000 nM of rivaroxaban delayed, but did not completely inhibit, thrombus formation. Dual antiplatelet agents weakly suppressed thrombus formation at both shear rates, but intensified the anticoagulant effects of dabigatran and rivaroxaban. The anticoagulant effects of dabigatran and rivaroxaban were also evaluated under static conditions using thrombin generation (TG) assay. In platelet-poor plasma, dabigatran at 250 and 500 nM efficiently prolonged the lag time (LT) and moderately reduce peak height (PH) of TG, whereas rivaroxaban at 250 nM efficiently prolonged LT and reduced PH of TG. In platelet-rich plasma, however, both anticoagulants efficiently delayed LT and reduced PH of TG. Our results suggest that dabigatran and rivaroxaban may exert distinct antithrombotic effects under flow conditions, particularly in combination with dual antiplatelet therapy.

## Introduction

Oral anticoagulants, dabigatran, a direct thrombin inhibitor (anti-IIa), and rivaroxaban, a direct factor Xa inhibitor (anti-Xa) represent novel therapeutic strategies for the prevention of deep vein thrombosis, and for the stroke prevention in atrial fibrillation [1]. In contrast to vitamin K antagonists, which typically require a titration using prothrombin time, these anticoagulants demonstrate predictable pharmacokinetics and anticoagulant responses, allowing for a fixed dosing regimen without routine monitoring [2], [3]. However, an option to assess the extent of anticoagulation is needed for patients with active bleeding associated with acute intestinal bleeding, trauma, and for those who require urgent invasive procedures [4], [5].

The combination of anticoagulant and antiplatelet therapies is a potential treatment strategy for acute coronary syndrome (ACS) because thrombin generation and fibrin formation can occur within the platelet thrombus during acute coronary events. It has been suggested that the addition of anti-IIa or anti-Xa agent to antiplatelet therapy may improve clinical outcomes after ACS [6]–[10]. However, these combination therapies are often associated with the increased risk of bleeding complications, implicating a relatively narrow therapeutic dose window [8]–[10]. It is, therefore, clinically important to individually assess residual hemostatic functions by testing anticoagulant and antiplatelet agents under the same conditions. However, this is not feasible using conventional platelet function assays and coagulation tests [11]. Some of the latter limitations may be overcome by evaluating fibrin-rich platelet thrombus formation under flow conditions [12].

In the present study, we evaluated the antithrombotic efficacies of dabigatran and rivaroxaban alone or in combination with antiplatelet agents by analyzing *in vitro* thrombus formation patterns under arterial and venous shear conditions in a flow-chamber system. A thrombin generation (TG) assay was performed in parallel to evaluate and characterize the effects of both anticoagulants under static conditions.

## Materials and Methods

### Materials

The cover and capillary chips used in the flow chamber system (**Fig. S1A**) were manufactured by Richell Corp. (Toyama, Japan). The following materials were obtained from commercial sources: porcine type I collagen (Nitta Gelatin, Inc., Osaka, Japan), tissue thromboplastin (Sysmex, Hyogo, Japan), fluorescein isothiocyanate (FITC)-conjugated mouse anti-human CD41 immunoglobulin G (IgG), and FITC-conjugated mouse IgG (Beckman Coulter, Miami, FL, USA), rabbit anti-human fibrinogen IgG (Dako, Tokyo, Japan), normal rabbit IgG (Santa Cruz Biotechnology, Santa Cruz, CA, USA), and Alexa594 (Invitrogen, Carlsbad, CA, USA).

Dabigatran and rivaroxaban were obtained from Toronto Research Chemicals, Inc. (Toronto, Canada). AR-C66096, a specific P_2_Y_12_-receptor antagonist, was obtained from Tocris Bioscience (Bristol, UK). For the TG assay, PPP-Reagent (with phospholipids), PRP-Reagent (without phospholipids), and FluCa-reagent, a fluorogenic substrate (Z-Gly-Gly-Arg-AMC) dissolved in HEPES buffer and calcium chloride, were purchased from Diagnostica Stago (Parsippany, NJ). Recombinant TF (r-TF) was purchased from Mitsubishi Chemical Medience (Tokyo, Japan). All other reagents were obtained from Wako Pure Chemicals (Osaka, Japan). Corn trypsin inhibitor (CTI) was prepared as reported previously [13].

### Blood samples

The study protocol was approved by the local ethics committee of Kinki University (Osaka, Japan), and informed written consent was obtained from 15 healthy, fasting volunteers (9 males, 6 females; mean age, 35.0±7.8 years). No subjects had taken any medication that might affect platelet function or coagulation in the preceding two weeks of blood collection. Blood samples were collected into plastic tubes containing 3.2% sodium citrate (Terumo, Tokyo, Japan), and were then mixed with CTI (final concentration, 50 µg mL^−1^). Citrated whole-blood samples were spiked with either dabigatran (250, 500, or 1000 nM) or rivaroxaban (250, 500, or 1000 nM) with or without the dual antiplatelet agents, aspirin (100 µM) and AR-C66096 (1 µM).

Dimethyl sulfoxide was used as the solvent (<0.1%, final concentration) for dabigatran, rivaroxaban and aspirin. This agent had no effect on flow chamber measurements at concentrations of up to 0.1%. The total volume of the added antithrombotic agents was less than 1% of the total blood volume.

### Preparation of microchips coated with collagen and thromboplastin

A section (15×15 mm) of each cover chip (**Fig. S1A**) was coated with type I collagen (1.5 mg mL^−1^) and tissue thromboplastin dissolved in 1 mM HCl. Both high thromboplastin (HTP; 100 µg mL^−1^) and low thromboplastin (LTP; 25 µg mL^−1^) were used to assess the impact of thromboplastin levels on the antithrombotic effects of dabigatran and rivaroxaban. The coated side of the cover chip was sealed with the capillary chip for 16 h at 60°C.

### Evaluation of thrombus formation under flow conditions

Thrombus formation under flow was evaluated using the T-TAS (Fujimori Kogyo, Kanagawa, Japan) as previously described [11]. The T-TAS system is equipped with a rectangular capillary (width, 300 µm; depth, 80 µm; length 15 mm), a pneumatic pump, and a flow pressure sensor (**Fig. S1B**). For the analysis, recalcified CTI-treated whole blood was perfused at 37°C through the pre-coated microcapillary-chamber (HTP or LTP as above). Flow pressure changes were monitored using the pressure transducer located upstream of the microcapillary chamber. During the perfusion of blood through the flow chamber, platelets and plasma coagulation pathways are simultaneously activated by collagen and tissue thromboplastin. Thrombus formation and breakdown within microcapillary results in flow disturbances, causing an increase and decrease of the pressure, respectively. At flow rates of 4 and 10 µl min^−1^, the initial wall shear rates were estimated to be 240 and 600 s^−1^, respectively according to the FLUENT program (Ansys Co., Ltd., Tokyo, Japan). These shear rates correspond to normal shear rates in large arteries and small veins (240 s^−1^), and those of medium-sized arteries (600 s^−1^) [14].

Endpoint parameters were as follows: (i) T_10_ (time to 10 kPa) represents the interval for the flow pressure to increase by 10 kPa from baseline due to the partial occlusion of the capillary; (ii) OT (occlusion time) represents the interval for the flow pressure to increase by 80 kPa from baseline due to the complete capillary occlusion; (iii) AUC_30_ (area under the curve for 30 min) represents the area under the flow pressure curve (below 80 kPa) for 30 min after the start of perfusion.

### Analysis of thrombi by confocal microscopy

Changes in the composition of thrombi due to dabigatran (500 nM) without/with antiplatelet agents were evaluated using immunostaining followed by confocal laser scanning microscopy. The thrombus specimen was collected from the mid-segment of the capillary, washed three times with phosphate-buffered saline (PBS), and then incubated with fluorescein isothiocyanate (FITC)-conjugated mouse anti-human CD41 (platelet glycoprotein IIb) IgG (1∶6 dilution) at room temperature for 15 min in the dark. FITC-conjugated mouse IgG was used as a control. After three washes with Tris-buffered saline containing 0.1% Triton X-100 (TBST), the thrombus was immobilized with OptiLyse C (Beckman Coulter, France) for 15 min at room temperature. After three additional washes with TBST, the sample was blocked for 1 h at room temperature with Block Ace (Yukijirushi, Osaka, Japan) containing 1 mg mL^−1^ normal goat IgG. The sample was then incubated with rabbit anti-human fibrinogen IgG (1∶99 dilution) labeled with Alexa 594 at room temperature for 30 min in the dark. Alexa 594-conjugated rabbit IgG was used as a control. The prepared thrombi specimens were examined using an LSM700 confocal microscope (Zeiss, Germany).

### Effects of antithrombotic agents on thrombin generation

Citrated blood samples were centrifuged at 125 x *g* and 1,760 x *g* for 10 min at room temperature to prepare platelet-rich plasma (PRP) and platelet-poor plasma (PPP), respectively. The platelet count in PRP was adjusted to 150×10^3^ µL^−1^ using autologous PPP. To prevent contact phase activation, PRP and PPP were mixed with CTI (final concentration, 50 µg mL^−1^). For TG experiments, 20 µl of PPP-Reagent (1pM or 5 pM) and PRP-Reagent were mixed with 80 µl PPP and PRP, respectively, in 96-well round-bottom microtiter plates (Thermo Fisher Scientific, Waltham, MA, USA). The final concentration of r-TF was 1 pM in PRP, whereas r-TF concentrations of 1 and 5 pM were tested for PPP. The plate was inserted into a Fluoroskan Ascent® fluorometer (ThermoLabsystem, Helsinki, Finland), and then warmed to 37°C for 5 min. TG experiments were initiated by automatically dispensing 20 µl of 2.5 mM fluorogenic substrate in 0.1 M CaCl_2_ to each well. Measurements were performed in duplicate for each experiment [15]. For each TG measurement, lag time (LT, min), peak thrombin concentration (PH, nM), and the endogenous thrombin potential (ETP, area under the TG curve, min x nM) were evaluated using the Thrombinoscope software (Thrombinoscope BV, Maastricht, Netherlands).

### Statistical analysis

Data are shown as the mean ± standard deviation (SD), unless otherwise indicated. Differences between groups were tested by one-way repeated ANOVA, followed by Tukey’s post-hoc test. To assess the efficacy of antiplatelet reagents, we calculated the relative ratio of OT values with and without antiplatelet agents (OT+_antiplatelet_/OT-_antiplatelet_) for dabigatran and rivaroxaban. The measured OT ratios at different concentrations were averaged to assess the difference between dabigatran and rivaroxaban. All statistical analysis were performed using Prism version 5.02 software (GraphPad Software, CA, USA). A *P* value of <0.05 was considered statistically significant.

## Results

### Effects of tissue thromboplastin levels on thrombus formation under flow conditions

**Figure 1:**
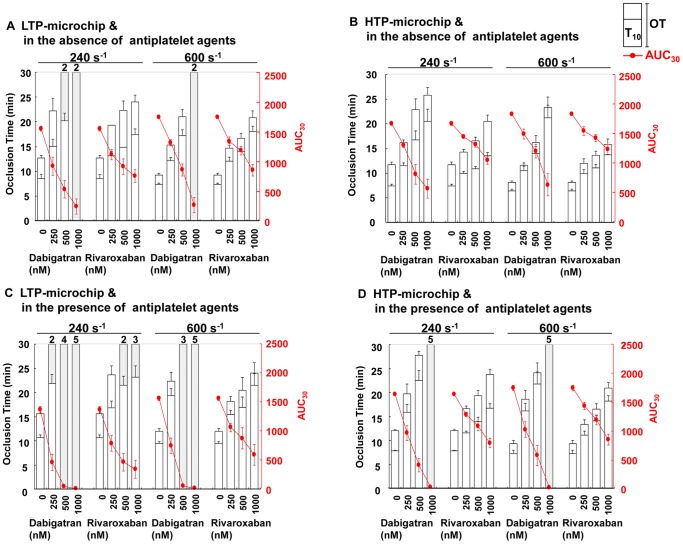
Effects of Dabigatran and Rivaroxaban on Thrombus Formation under Flow Conditions. The effects of dabigatran and rivaroxaban on thrombus formation in the absence (**panels A and B**) and presence of aspirin/AR-C66096 (**panels C and D**) were evaluated using T-TAS. Thrombus formation inside LTP (low thromboplastin) microchips (**panels A and C**) and HTP (high thromboplastin) microchips (**panels B and D**). Data (n = 5) are shown as mean values. Shaded boxes indicate the values for T_10_ and/or OT exceeding 30 min, and the frequency of such measurements is indicated above each box. AUC_30_, area under the curve for 30 min; OT, occlusion time; T_10_, time to 10 kPa.

At a shear rate of 240 s^−1^, the onset (T_10_) and capillary occlusion (OT) for control whole-blood samples perfused over LTP-microchips were 8.56±0.79 min and 12.71±0.56 min, respectively (**Fig. 1A**). For HTP-microchips at 240 s^−1^, the T_10_ and OT values were 7.44±0.32 min and 11.71±0.52 min (**Fig. 1B**). Thrombus formation was accelerated in the control blood samples at a shear rate of 600 s^−1^. T_10_ and OT values for LTP-microchips were 7.36±0.24 min and 9.26±0.37 min, respectively, and those for HTP-microchips were 6.43±0.30 min and 8.12±0.34 min, respectively, at 600 s^−1^ (**Fig. 1A, B**).

### Antithrombotic effects of dabigatran and rivaroxaban under flow conditions

Dabigatran and rivaroxaban suppressed thrombus formation at both examined shear rates in a concentration-dependent manner for LTP- and HTP-microchips (**Fig. 1A, B**). Using LTP-microchips, dabigatran at the concentration of 1000 nM almost completely suppressed thrombus formation, as indicated by prolonged OT and lower AUC, at both 240 and 600 s^−1^, whereas 1000 nM rivaroxaban only moderately suppressed thrombus formation (**Fig. 1A**). Compared to LTP-microchips, the prolongations of T_10_ and OT by both antithrombotic agents were less extensive when HTP-microchips were used. In the whole blood treated with 500 nM dabigatran, the OT values for LTP- and HTP-microchips were prolonged by 2.36- and 1.95-fold, respectively, at 240 s^−1^, and by 2.27- and 1.99-fold, respectively, at 600 s^−1^. Similarly, 500 nM rivaroxaban increased OT values for LTP- and HTP-microchips by 1.75- and 1.41-fold, respectively, at 240 s^−1^, and by 1.80- and 1.68-fold, respectively, at 600 s^−1^ (**Fig. 1A, B**). Taken together, dabigatran appeared to inhibit thrombus formation more potently than rivaroxaban regardless of the tissue thromboplastin levels or shear rates.

Dual platelet inhibition using aspirin and AR-C66096 only weakly suppressed thrombus formation, but enhanced the antithrombotic efficacy of both dabigatran and rivaroxaban (**Fig. 1C, D**). In terms of the ratio of OT (OT_+antiplatelet_/OT_-antiplatelet_), dual anti-platelet agents increased OT values of dabigatran- or rivaroxaban-treated blood by 1.48-fold and 1.20-fold, respectively, as an average under all experimental conditions (at high and low shear rates and at HTP and LTP concentrations). This indicates that the antithrombotic activity of dabigatran is more affected than that of rivaroxaban in the presence of aspirin and AR-C66096 under arterial flow (**Video S1**).

### Analysis of thrombi by confocal microscopy

**Figure 2:**
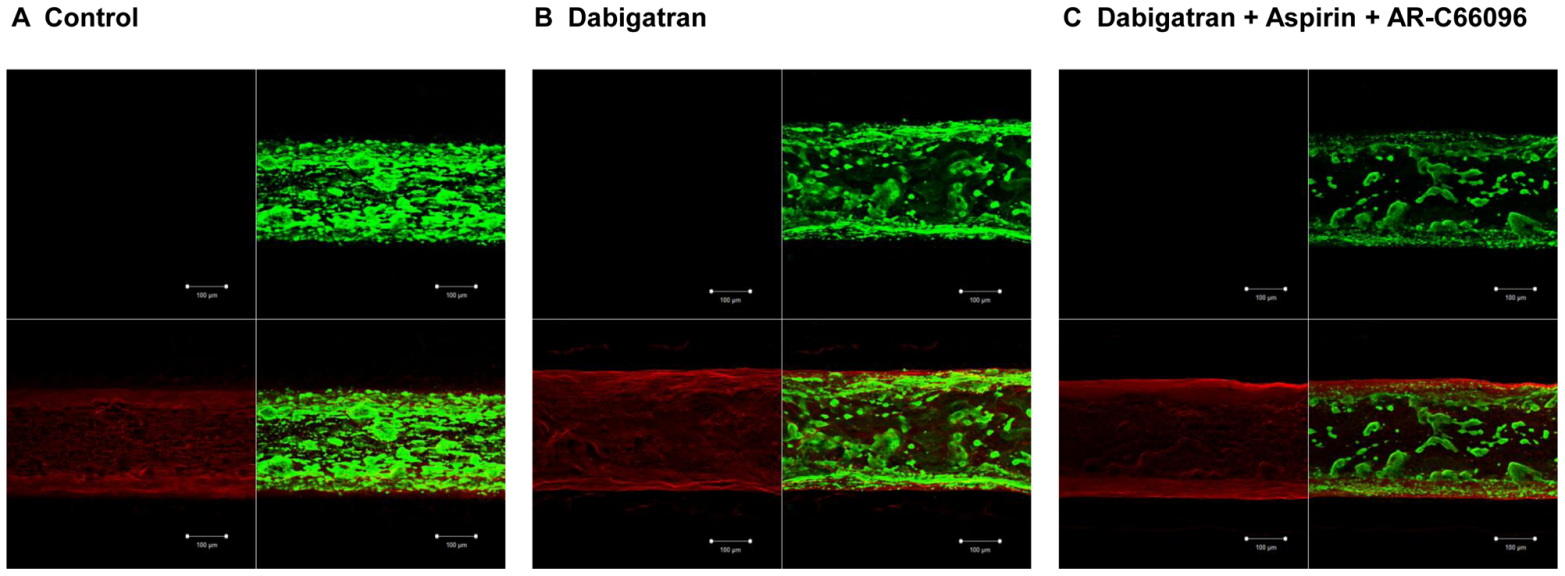
Confocal Images of Immunostained Platelets and Fibrin. Immunostained thrombi in non-treated control blood (**panel A**), and in the blood pre-treated with dabigatran (**panel B**), or dabigatran, aspirin, and AR-C66096 (AR-C) (**panel C**) after perfusion over the HTP (high thromboplastin) microchips at 600 s^−1^ are shown. For **panels A, B and C**, isotype-matched control (upper left), platelet GPIIb stained by anti-CD41-FITC (green; upper right), fibrin stained by anti-fibrinogen-Alexa 594 (red; lower left), and a merged image (lower right) are shown.

The confocal images of thrombi formed at a shear rate of 600 s^−1^ indicated progressive decreases in the intra-luminal thrombus extension by dabigatran and dabigatran plus dual antiplatelet inhibitions (**Fig. 2**).

### Effects of dabigatran and rivaroxaban on endogenous TG

**Figure 3:**
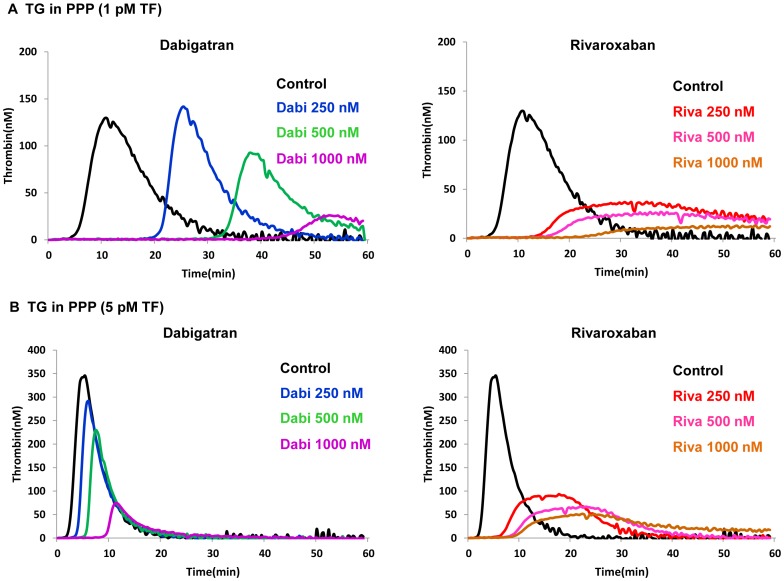
Effects of Dabigatran and Rivaroxaban on TG in PPP. The inhibitory effects of dabigatran (Dabi) and rivaroxaban (Riva) on thrombin generation (TG) in platelet-poor plasma were evaluated using the Thrombinoscope™. **Panel A:** TG triggered by 1 pM recombinant tissue factor, and **Panel B:** TG triggered by 5 pM recombinant tissue factor. Typical TG patterns reflecting distinctive efficacies of dabigatran and rivaroxaban are shown.

**(i) TG in PPP.** In TG measurements using PPP in the presence of 1 pM r-TF, dabigatran at 500 nM prolonged LT and TTP by 5.24- and 3.29-fold, respectively, and decreased PH by 25.0%, compared to the untreated control (**Fig. 3A** **,** **Table 1**). Under the same conditions, rivaroxaban at 500 nM prolonged LT and TTP by 3.05- and 2.88-fold, respectively, and reduced PH by 83.0%, of that of the control (**Fig. 3A** **,** **Table 1**). Similarly, when added to PPP containing 5 pM r-TF, dabigatran at 500 nM prolonged LT and TTP by 2.89- and 1.88-fold, respectively, compared to the untreated control, and moderately decreased PH by 24.1%. In contrast, rivaroxaban at 500 nM prolonged LT and TTP by 3.44- and 4.25-fold, respectively, compared to the control and reduced PH by 80.0% (**Fig. 3B** **,** **Table 2**). Taken together, these results demonstrate that rivaroxaban exerts extensive PH reduction at lower concentrations. This inhibition follows the saturable Michaelis-Menten kinetics, while dabigatran demonstrates more linear reduction in PH at both thromboplastin levels (**Fig. 3A, 3B**). LT and TTP were prolonged in a concentration-dependent manner, and this effect did not depend on thromboplastin level for rivaroxaban.

**Table 1:** Effects of Dabigatran and Rivaroxaban on TG in PPP (low tissue factor).

Treatment	LT (min)	TTP (min)	PH (nM)	ETP (min x nM)
Control	5.8±0.6	10.6±0.4	117.1±21.2	1453±199
Dabi 250 nM	18.7±2.1^***^	22.4±2.2^***^	141.0±24.9	1352±197
Dabi 500 nM	30.4±3.1^***^	34.9±3.3^***^	87.8±10.7^*^	832±63^**^ §
Dabi 1000 nM	40.5±6.2^***^	49.5±6.7^***^	22.6±7.2^**^	118±83^**^ §
Riva 250 nM	15.5±0.3^***^	31.8±1.2^***^	34.1±1.0^**^	621±16^**^ §
Riva 500 nM	17.7±0.5^***^	30.5±5.3^**^	19.9±8.2^**^	343±86^***^ §
Riva 1000 nM	22.7±2.2^***^	50.6±3.3^***^	12.6±1.8^***^	63±53^***^ §

TG, thrombin generation triggered by 1 pM recombinant tissue factor; PPP, platelet-poor plasma; Dabi, dabigatran; Riva, rivaroxaban; LT, lag time; TTP, time to peak of TG; PH, peak height of TG; ETP, endogenous thrombin generation potential.

Data are shown as the mean ± SD. * *P*<0.05, ** *P*<0.01, and *** *P*<0.001 *vs.* control.

§ indicates that, since TG curves did not come down to the baseline within 60 min, ETP values were calculated by setting the start tail at 60min.

**Table 2:** Effects of Dabigatran and Rivaroxaban on TG in PPP (high tissue factor).

Treatment	LT (min)	TTP (min)	PH (nM)	ETP (min x nM)
Control	2.7±0.4	5.2±0.5	329.4±24.7	1942±152
Dabi 250 nM	5.6±1.0^**^	7.7±1.1^**^	313.0±30.9	1792±207
Dabi 500 nM	7.8±1.3^***^	9.8±1.2^***^	250.1±27.4^***^	1307±147^***^
Dabi 1000 nM	11.6±1.8^***^	13.8±1.7^***^	85.3±12.4^***^	574±54^***^
Riva 250 nM	7.9±0.5^***^	18.6±0.9^***^	89.0±3.6^***^	1616±21^*^
Riva 500 nM	9.3±0.3^***^	22.1±1.1^***^	66.0±1.7^***^	1436±32^**^
Riva 1000 nM	10.6±0.4^***^	25.1±1.1^***^	48.3±1.9^***^	982±44^***^ §

TG, thrombin generation triggered by 5 pM recombinant tissue factor; PPP, platelet-poor plasma; Dabi, dabigatran; Riva, rivaroxaban; LT, lag time; TTP, time to peak of TG; PH, peak height of TG; ETP, endogenous thrombin generation potential.

Data are shown as the mean ± SD. * *P*<0.05, ** *P*<0.01, and *** *P*<0.001 *vs.* control.

§ indicates that, since TG curves did not come down to the baseline within 60 min, ETP values were calculated by setting the start tail at 60min.

**Figure 4:**
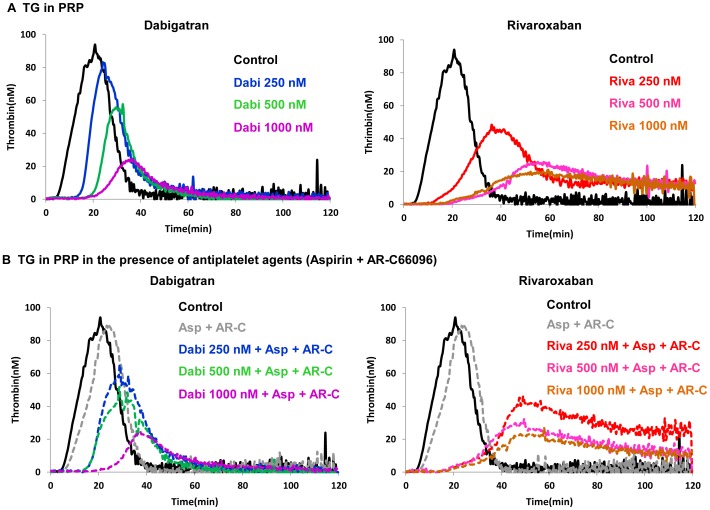
Effects of Dabigatran and Rivaroxaban on TG in PRP. The inhibitory effects of dabigatran (Dabi) and rivaroxaban (Riva) on thrombin generation (TG) in platelet-rich plasma in the absence (**panel A**) and presence of 100 µM aspirin (Asp) together with 1 µM AR-C66096 (AR-C) (**panel B**) were calculated using Thrombinoscope™. Typical TG patterns obtained in one of the experiments are shown.

**(ii) TG in PRP.** In the TG assay using PRP, both dabigatran and rivaroxaban prolonged LT and TTP, and reduced PH in a concentration-dependent manner (Fig. 4). Relative to the untreated control, dabigatran and rivaroxaban at 500 nM prolonged both LT (3.18- and 3.56-fold, respectively) and TTP (1.60- and 2.53-fold, respectively) and decreased PH by 42.5% and 67.1%, respectively (Fig. 4A, Table 3). The ETP also had tendency to decrease, but accurate calculation for this effect was impossible at some concentrations. After addition of aspirin and AR-C66096 to PRP, LT and TTP values were minimally prolonged without essential changes in PH value (Fig. 4B, Table 3). Similarly, no statistically significant changes in any of the TG parameters were observed by the addition of aspirin and AR-C66096 to the dabigatran or rivaroxaban treatment of PRP (Fig. 4B, Table 3).

**Table 3:** Effects of Dabigatran and Rivaroxaban on TG in PRP in the presence and absence of Aspirin and AR-C66096.

Treatment	LT (min)	TTP (min)	PH (nM)	ETP (min x nM)
Control	6.8±0.8	19.7±1.8	92.1±23.7	1547±297
Dabi 250 nM	18.6±5.4^***^	27.9±7.3	69.2±20.6	1286±352§
Dabi 500 nM	21.6±5.3^***^	31.5±6.8	53.0±5.8***	1012±120
Dabi 1000 nM	30.2±5.6^***^	41.8±4.8^***^	23.0±6.9***	565±136^***^ §
Asp + AR-C	9.4±3.2	23.3±2.8	91.2±29.5	1504±252
Dabi 250 nM + Asp+AR-C	21.1±4.5^***^	35.7±8.1	52.2±13.6***	1195±365
Dabi 500 nM + Asp+AR-C	26.2±10.2^***^	38.0±10.6	47.8±9.0***	1101±154§
Dabi 1000 nM + Asp+AR-C	35.1±6.3^***^	49.9±8.2^***^	22.0±8.4^***^	465±197^***^ §
Riva 250 nM	20.1±4.0^***^	39.0±3.6^**^	44.7±6.8^***^	1142±229§
Riva 500 nM	24.2±6.2^***^	49.8±10.5^***^	30.3±7.8^***^	980±351§
Riva 1000 nM	31.2±9.8^***^	68.7±17.1^***^	20.8±7.7^***^	543±320^***^ §
Riva 250 nM +Asp+AR-C	22.2±3.8^***^	45.7±5.9^***^	44.4±9.3^***^	1156±794§
Riva 500 nM + Asp+AR-C	26.7±5.3^***^	56.5±12.2^***^	31.2±8.0^***^	901±278^*^ §
Riva 1000 nM + Asp+AR-C	30.8±7.0^***^	78.6±24.4^***^	18.1±9.3^***^	480±410^***^ §

TG, thrombin generation triggered by 1 pM recombinant tissue factor; PRP, platelet-rich plasma; Asp, aspirin 100 µM; AR-C66096, 1 µM; Dabi, dabigatran; Riva, rivaroxaban; LT, lag time; TTP, time to peak of TG; PH, peak height of TG; ETP, endogenous thrombin generation potential.

Data are shown as the mean ± SD. ^*^ *P*<0.05, ^**^ *P*<0.01, and ^***^ *P*<0.001 *vs.* Asp+AR-C.

§ indicates that, since TG curves did not come down to the baseline within 120 min, ETP values were calculated by setting the start tail at 120min.

## Discussion

Our present data demonstrate that dabigatran and rivaroxaban possess distinctive antithrombotic properties under arterial and venous shear conditions. Anticoagulant activities of both drugs appear to be influenced by localized tissue thromboplastin, and by the presence of antiplatelet agents. Notably, antithrombotic efficacies of dabigatran and rivaroxaban under flow conditions markedly differ from those of static coagulation tests, particularly in the presence of antiplatelet agents.

In the microchip-based flow chamber analysis, dabigatran efficiently reduced fibrin-rich platelet thrombus formation, and delayed capillary occlusion (OT) more extensively than rivaroxaban at both tested shear rates (**Fig. 1A, B**). The formation of fibrin-rich platelet thrombi was only weakly inhibited by aspirin and AR-C66096 in non-anticoagulated whole blood; however, these two antiplatelet agents enhanced the antithrombotic efficacies of both anticoagulants (**Fig. 1C, D**). Dabigatran exhibited more potent antithrombotic activity in combination with aspirin and AR-C66096 than that of rivaroxaban under arterial shear flow (600 s^−1^). These findings are in contrast with the anticoagulant efficacies of dabigatran and rivaroxaban shown in the TG measurements. In plasma without platelets, low concentrations of dabigatran decreased PH in a concentration-dependent manner more slowly than corresponding concentrations of rivaroxaban (**Fig. 3A, B**). Our TG data in PPP are in agreement with previously published data by Furugohri, *et al.* [**16**], and Marlu, et al. [17] Both studies demonstrated more extensive suppression of peak TG with anti-Xa agents (edoxaban or rivaroxaban) compared to the anti-IIa agents (melagatran, lepirudin or dabigatran).

Conversely, our TG data in platelet-rich plasma demonstrated that dabigatran and rivaroxaban similarly suppressed TG peak levels (**Fig. 4**). When TG is triggered without exogenous phospholipids, thrombin-mediated platelet activation becomes an important source of phospholipids. Rivaroxaban inhibits FXa and FXa-FVa complexes on activated platelets [18], whereas dabigatran impairs platelet-surface mediated TG by blocking platelet activation via PAR1 or PAR4 [19]. Thrombin-mediated platelet activation is more critical under flow conditions, particularly in the presence of antiplatelet agents, as our flow-chamber experiments demonstrated more extensive thrombus suppression using dabigatran than rivaroxaban.

Our present confocal microscopy analysis was performed to characterize different compositions of thrombi formed in the presence dabigatran alone or in combination with dual platelet inhibition with aspirin and AR-C66096. Intraluminal platelet deposition was decreased in the presence of dabigatran compared to non-anticoagulated control (**Fig. 2A, B**), and further reduction of platelet deposition was observed with dabigatran and dual antiplatelet agents (**Fig. 2C**). Inhibiting collagen-induced platelet procoagulant activity was also demonstrated with anti-IIa bivalirudin within clinical concentrations (mean 2.7 µM) via PAR1 inhibition [20]. In our flow-chamber model, thromboplastin and collagen are initial triggers for thrombus formation, but sequential activations of platelet receptors for collagen, TXA_2_, ADP and PARs are also reflected [21], [22]. In contrast to the improved antithrombotic efficacies of dabigatran and rivaroxaban in combination with antiplatelet agents under arterial shear, our static TG model in PRP failed to exhibit the combined effects of anticoagulant and antiplatelet agents (**Fig. 4** **,** **Table 3**).

In our flow-chamber model, exposure to thromboplastin (extrinsic pathway) and collagen is limited to the platelets and plasma flowing adjacent to the capillary wall. Subsequent thrombus growth depends on local recruitment and activation of platelets, and thrombin-mediated feedback activation of the intrinsic pathway [23]. Procoagulant activities of FXa and thrombin are also limited by blood flow, which prevent their efficient interaction with substrates [24]. In static plasma coagulation model, initial TG is highly dependent on TF level, and FXa is its limiting step [25]. The contrasting data between flow and static conditions qualitatively confirm that low PH in static TG is not a prerequisite for effective antithrombotic activity *in vivo*.

Our *in vitro* data in the flow chamber experiments are corroborated by the increased antithrombotic, and potentially hemorrhagic effects of dual antiplatelet therapy with anti-IIa or anti-Xa agents in patients with ACS [8]–[10]. The mono-antiplatelet therapy with aspirin combined with an anti-IIa, ximelagatran was reported to decrease the recurrent cardiovascular events without a major increase in bleeding events [7]. Conversely, the addition of dabigatran to dual antiplatelet therapy increased minor and major bleeding complications, but it did not improve clinical outcomes of the patients with acute coronary syndromes [9]. The combination of low-dose rivaroxaban (2.5 mg twice daily) and antiplatelet therapy significantly reduced death from cardiovascular causes and myocardial infarction in patients with recent ACS [10], but the incidence of bleeding complications, including intracranical hemorrhage, was increased [10].

A few limitations of the present study should be mentioned. First, endothelial cells are excluded form the microchip-based flow chamber system, and therefore it is not feasible to evaluate interactions between anticoagulants and endothelial elements (*e.g*., thrombomodulin) [16]. Second, subendothelial TF expression varies among vascular beds, a property that may in part explain the increased gastrointestinal bleeding, but the decreased incidence of hemorrhagic stroke, associated with dabigatran relative to warfarin [26]. Although two different concentrations of thromboplastin were tested in our study, our *in vitro* results cannot be directly inferred to clinical efficacy or hemorrhagic risk.

Increasing numbers of antiplatelet and anticoagulant agents are becoming available for clinical use [5], [27], but few monitoring techniques exist for the assessment of various antithrombotic agents and their combinations [28]. The ability of microchip-based flow chamber systems to rapidly evaluate and characterize antithrombotic efficacies of drug combinations under flow conditions is potentially useful in the dose adjustment, and the selection of optimal agents. Future clinical studies using ex vivo blood samples collected from patients receiving antithrombotic therapies are warranted to correlate pertinent analytical parameters of the flow system with clinical thrombosis and/or bleeding outcomes.

## Supporting Information

Figure S1 **Schematic diagrams of the microchip flow chamber system.** (A) The microchip. (B) The analytical set up.
(TIF)

Video S1 **Video images of the thrombus formation process.** Thrombus formation in the presence and absence of dabigatran, Aspirin and AR-C66096.
(MP4)
