# Supplementary figures and images for: Comparative Evaluation of Direct Thrombin and Factor Xa Inhibitors with Antiplatelet Agents under Flow and Static Conditions: An In Vitro Flow Chamber Model
Source: PLoS One. 2014 Jan 31;9(1):e86491. doi: 10.1371/journal.pone.0086491 (PMC3908954; doi:10.1371/journal.pone.0086491)

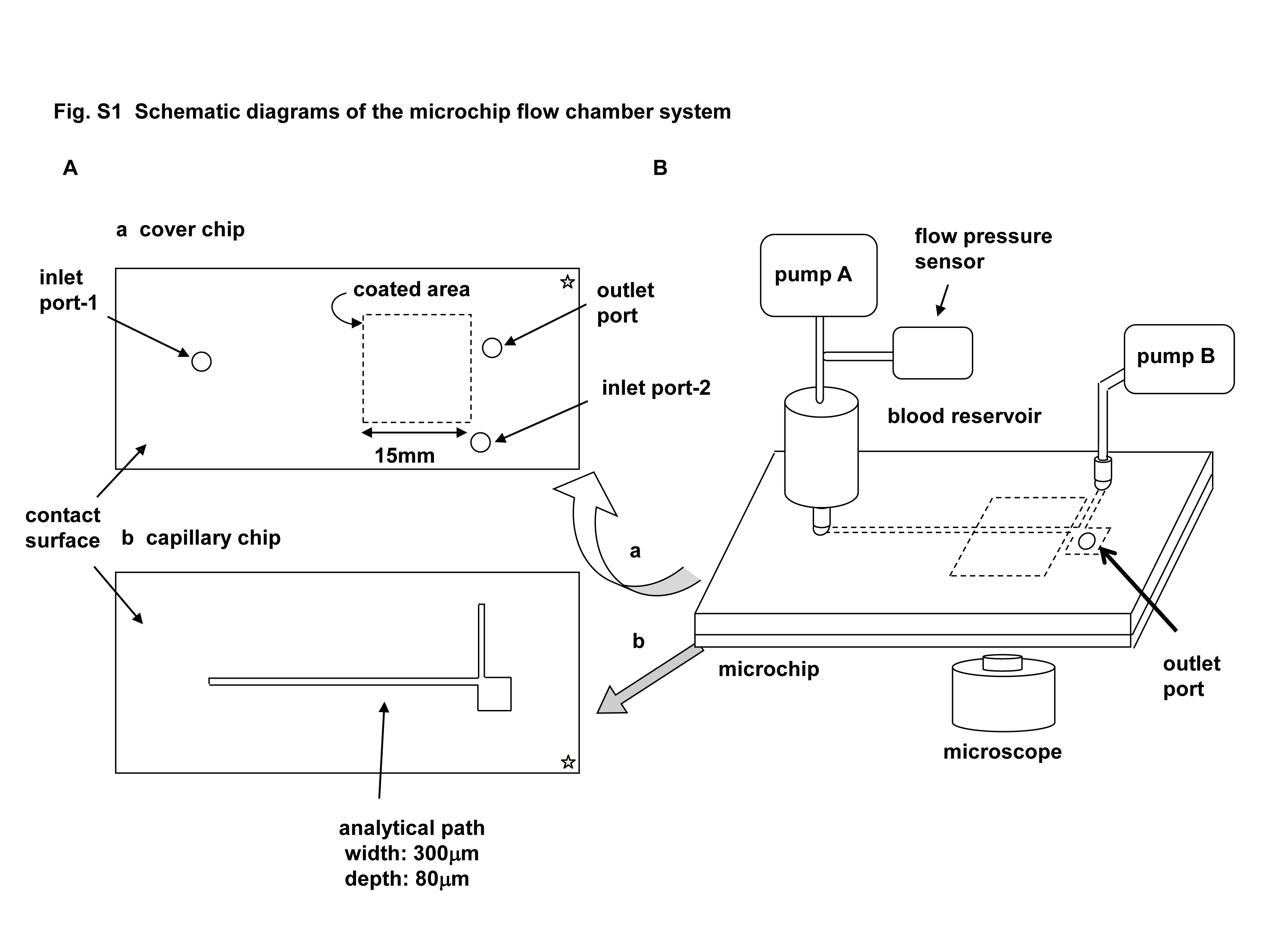

Supplement: Figure S1 — Schematic diagrams of the microchip flow chamber system. (A) The microchip. (B) The analytical set up. (TIF) [file pone.0086491.s001.tif]
